# Bimodal Copulatory Locomotion and Ritualized Male–Male Combat in the Amazonian Lancehead (*Bothrops atrox*): Linking Sexual Size Dimorphism to Reproductive Mechanics and Sexual Conflict

**DOI:** 10.3390/ani16213419

**Published:** 2026-10-29

**Authors:** Ligia G. S. Amorim, Vincent Prémel, Lucas Aosf, Karina Nunes Kasperoviczus, Selma Maria de Almeida-Santos

**Affiliations:** 1 Instituto de Biociências, Letras e Ciências Exatas, Universidade Estadual Paulista (UNESP), São José do Rio Preto 15054-000, SP, Brazil; selma.santos@butantan.gov.br; 2 Laboratório de Ecologia e Evolução, Instituto Butantan, São Paulo 05503-900, SP, Brazil; karina.nunes@fundacaobutantan.org.br; 3 Institute of Systematics, Evolution, Biodiversity, École Pratique des Hautes Études, 75014 Paris, France; v.premel@yahoo.fr; 4 Instituto de Manejo da Biodiversidade (IMBio), Guaratinguetá 12505-250, SP, Brazil; lucasaosf@gmail.com

**Keywords:** Viperidae, Neotropical snakes, mating systems, ethogram, Guiana Shield, behavioral ecology, female control, hemipenial morphology

## Abstract

Reproductive behaviors in secretive Neotropical pitvipers are rarely documented under natural conditions, limiting empirical assessments of sexual selection, locomotor mechanics, and mating systems. Here, we present focal videography and photographic documentation of free-ranging Amazonian Lanceheads (*Bothrops atrox*) from coastal French Guiana during the rainy season. A prolonged copulation (~3 h) revealed a remarkable bimodal locomotor sequence: the pair moved from terrestrial litter across a 20 m-wide forest stream and back to land without cloacal separation. Video analysis demonstrates female-driven locomotion, wherein the substantially larger female pulls the coupled male through the water column against his counter-directional swimming movements. This behavior illustrates female locomotor dominance under sexual size dimorphism and the widely mechanical resilience of the spine-bearing crotaline hemipenis during forced aquatic displacement. Sequential photographic documentation captures ritualized male–male combat with synchronized vertical displays, anterior-trunk swaying, neck hooking, and topping attempts, confirming that stereotypic motor patterns described in western Amazonia are conserved in eastern Guiana Shield populations. These postural dynamics represent transient physical contests over spatial priority rather than permanent dominance hierarchies. Together, this evidence indicates that empirical validation for female behavioral control during copulation and expands the geographic framework supporting the prolonged mate-searching polygyny hypothesis in *B. atrox*.

## Simple Summary

The knowledge about wild snakes’ reproduction presents a wide challenge, since individuals are difficult to observe in nature. In the present study, we recorded rare video and photographic evidence of Amazonian Lancehead pitvipers during their breeding season in French Guiana. During a three-hour mating event, a larger female moved from land into a 20 m-wide stream and swam across while pulling the attached male, who resisted by swimming in the opposite direction, demonstrating that females dominate movement during mating. In addition, male reproductive organs could endure significant physical stress, without separating. We also documented two males engaging in an upright wrestling match to compete for mates, proving that this competitive behavior is shared across different Amazonian populations. These discoveries provide important insights into animal behavior, physical performance, such as the evolution of mating strategies, helping scientists to obtain a better understanding and conserve tropical wildlife and rainforest ecosystems.

## 1. Introduction

Understanding how reproductive behaviors, including courtship, copulation, and intrasexual agonistic interactions, influence the reproductive biology of the species is a central question in evolutionary ecology. In solitary, cryptic ambush predators such as viperid snakes, direct field observations of these interactions remain exceptionally scarce [1,2,3,4]. Consequently, theoretical models of snake mating systems—such as prolonged mate-searching polygyny, female defense, and scramble competition—have largely been inferred from captive observations, morphological proxies, or indirect radiotelemetry movement patterns rather than continuous behavioral recordings in the wild [5,6].

In Neotropical pitvipers (genus *Bothrops*), reproductive cycles are closely tied to regional rainfall regimes [1]. In Amazonian populations of the Common Lancehead (*Bothrops atrox*), vitellogenesis in females and sexual segment of the kidney (SSK) hypertrophy in males peak during the rainy season, synchronizing mating activity between January and May [7]. Females of *B. atrox* display pronounced sedentary habits and cryptic ambush foraging, whereas adult reproductive males substantially increase their daily movement rates during the wet season to detect female scent trails and pheromones, which indicates a higher incidence during the mating period [7,8,9]. This marked behavioral divergence in spatial mobility is the operational foundation of the prolonged mate-searching polygyny hypothesis [4,10]. Under this system, searching males that encounter one another in the vicinity of a receptive female engage in ritualized combat to guarantee priority copulatory access [4,5,6].

Despite the broad geographic distribution of *B. atrox* across the Amazon Basin as well as the Guiana Shield [11], natural behavioral records remain heavily clustered in central and western Brazilian Amazonia [4,7,8]. Field documentation of male–male combat in free-ranging *B. atrox* was unknown until the recent record in Acre, western Brazilian Amazonia [4]. Furthermore, while copulation has occasionally been observed in nature and semi-natural conditions [7,12], the biomechanical forces governing pair locomotion during prolonged copula, intersexual physical conflict, and the substrate transitions traversed by coupled pairs have never been examined dynamically.

Here, using continuous focal videography and detailed photographic documentation coupled with standardized ethological descriptions, we present novel insights into the reproductive interactions of free-ranging *B. atrox* in coastal French Guiana, comprising two mating events, one advanced courtship, and one male–male combat interaction. Given the environmental differences between Amazonian and Guianan locations, we aim to verify if behavioral traits are conserved. We specifically investigate: (1) the locomotor mechanics and intersexual physical conflict during an extended copulation involving a terrestrial-to-aquatic habitat transition; and (2) the motor patterns of male–male combat, assessing behavioral conservation across geographically disjunct populations and testing the behavioral predictions of the mate-searching polygyny hypothesis. Overall, our findings reveal that female-biased sexual size dimorphism grants total locomotor control to the female during copulation—allowing transit across aquatic barriers despite male counter-traction—and confirm that ritualized combat behaviors are geographically conserved across Amazonian lineages, providing empirical support for female behavioral control and expanding the geographic framework of the prolonged mate-searching polygyny hypothesis in *B. atrox*.

## 2. Materials and Methods

### 2.1. Study Sites and Observation Protocols

**Figure 1:**
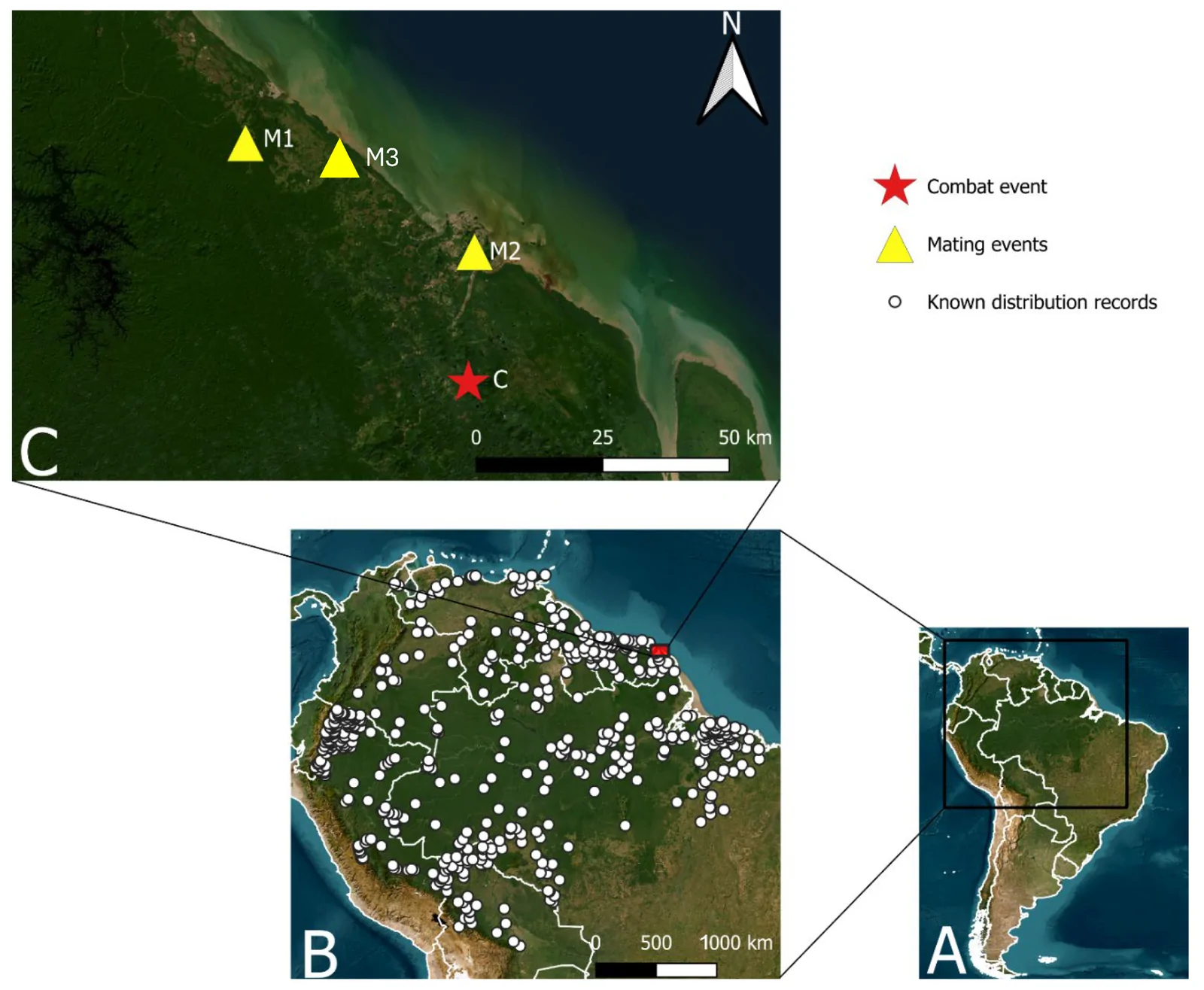
Geographic distribution and observation sites of *Bothrops atrox* reproductive interactions in coastal French Guiana. (**A**) Overview map of South America showing the location of the study region. (**B**) Broad geographic distribution of *B. atrox* in northern South America based on verified spatial occurrence data [11] (white circles), avoiding overestimation artifacts of minimum convex polygons. (**C**) Detailed map of coastal French Guiana depicting specific observation sites: mating events (yellow triangles: M1, Maré de Fourgassié, Roura [bimodal copulation]; M2, Chemin de l’Habitation Vidal, Rémire-Montjoly [diurnal copulation]; M3, Camp Cariacou, Kourou [advanced courtship]) and male–male combat (red star: C, Montagne des Singes, Kourou).

Field observations of free-ranging *Bothrops atrox* were conducted opportunistically between February 2024 and April 2026 at four distinct localities within primary and secondary lowland rainforests of coastal French Guiana, representing the northeastern margin of the species’ geographic range [11] (Figure 1; Table 1): Maré de Fourgassié, Roura (4°38′37.3″ N, 52°17′57.5″ W; 242 m a.s.l.); Montagne des Singes, Kourou (5°04′15.2″ N, 52°41′48.8″ W; 85 m a.s.l.); Chemin de l’Habitation Vidal, Rémire-Montjoly (4°52′37.3″ N, 52°17′19.7″ W; 15 m a.s.l.); and Camp Cariacou, Kourou (4°58′36.5″ N, 52°42′46.1″ W). Microclimatic variables (ambient temperature and relative humidity) were recorded in situ at the encounter microhabitats using a calibrated digital thermo-hygrometer (precision of 0.1 °C). Geographic coordinates and elevations were acquired via a handheld GPS unit (WGS84 datum).

Behavioral interactions were documented using digital imaging systems (Sony A7III—90 mm macro lens, Panasonic DMC-FZ2000 and iPhone 15 Pro for focal videography and telephoto photographic systems). Field observations followed an ad libitum event sampling regime, combining continuous focal animal video recording for the primary mating event with sequential telephoto photographic documentation for subsequent encounters [13]. Observers maintained an unobtrusive distance (at least 3 m) to prevent anthropogenic disturbance of ongoing motor displays. The copulatory video sequence was archived as Supplementary Material (Video S1) and analyzed frame-by-frame using VLC media player (v. 3.0.18), while sequential photographic series were examined chronologically to construct detailed ethological descriptions using standard crotaline behavioral terminology [2,4]. Calling anuran species heard during the aquatic phase of the Roura mating observation were identified acoustically from their advertisement calls by a specialist.

### 2.2. Sex Determination and Ethical Declarations

During mating interactions records, sex was confirmed unambiguously by direct visual observation of cloacal coupling, relative body orientation, and hemipenis eversion. In *B. atrox*, sexual size dimorphism (SSD) is markedly female-biased, with adult females attaining substantially greater snout–vent length (SVL) and body mass than conspecific males [7]. During male–male combat records, given the strictly non-invasive protocol, the sex of the combatants was not anatomically confirmed (e.g., via cloacal probing) but inferred robustly based on the stereotyped vertical displays (topping and swaying), which are male-exclusive reproductive behaviors in crotalines [2,4,6]. For this combat event, continuous individual identification (Male 1 vs. Male 2) was maintained via natural phenotypic markers tracking, such as slight body size differences and subtle variations in the dorsal pattern.

Field observations were strictly non-invasive, relying exclusively on remote optical recording without physical handling or capturing the animals. All procedures complied with the ethical guidelines for field research on live amphibians and reptiles established by the Herpetological Animal Care and Use Committee (HACC) of the American Society of Ichthyologists and Herpetologists and adhered to the environmental regulations of French Guiana (Direction de l’Environnement, de l’Aménagement et du Logement—DEAL).

## 3. Results

### 3.1. Event 1: Prolonged Mating, Bimodal Locomotion, and Hemipenial Retraction (Roura)

**Figure 2:**
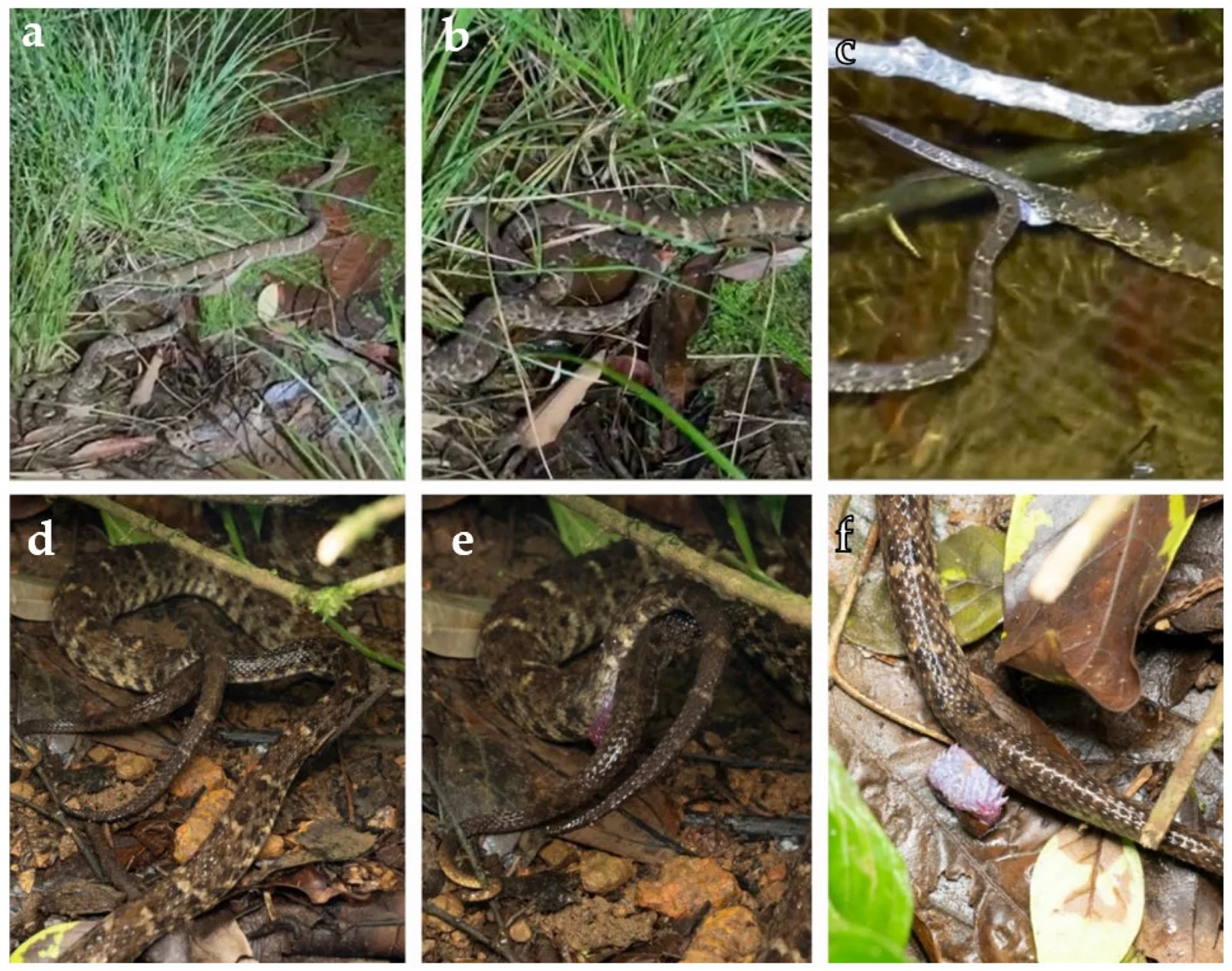
Bimodal copulatory locomotion and mechanical resilience under female-biased sexual size dimorphism in *B. atrox* (Event 1; Roura). (**a**,**b**) The coupled pair on terrestrial leaf litter, with the larger female positioned anteriorly. (**c**) The female actively swimming across a 20 m-wide forest stream, pulling the coupled male as hydrodynamic drag against his counter-directional swimming movements. (**d**) The pair returning to terrestrial substrate while maintaining cloacal locking. (**e**) Mechanical separation following sustained female forward movement, showing the male’s everted hemipenis. (**f**) The male remaining stationary on the ground during the active muscular retraction of the hemipenis prior to retreat.

On February 16th of 2024, at 20:00 h, an adult pair of *B. atrox* was discovered in copula on leaf litter within terra firme forest (i.e., non-seasonally flooded upland Amazonian rainforest) at Maré de Fourgassié, Roura (Tair = 24.2 °C, relative humidity ~100%) (Table 1). The female was positioned anteriorly, lying prone in the litter, while the smaller male remained coiled with his posterior trunk aligned beneath the female’s tail base (Figure 2a,b).

The pair was continuously monitored from 20:00 h until 21:00 h, left undisturbed, and re-encountered at 23:00 h approximately 15 m from the original site, remaining continuously coupled. At this stage, the female initiated directed crawling toward the margin of a slow-moving forest stream (igarapé; width ~20 m, maximum depth ~1 m). As documented in Supplementary Material (ESM Video S1), the female entered the water and swam across the stream surface using lateral undulation. The coupled male did not swim cooperatively; instead, he aligned his body in the opposite direction and executed vigorous reverse swimming strokes possibly indicating an effort to disengage or anchor himself against the current. Due to the female’s markedly larger body mass, her forward propulsive thrust completely overpowered the male’s resistance, towing him as a passive hydrodynamic drag across the entire width of the stream (Figure 2c; ESM Video S1).

Despite substantial hydrodynamic shear forces and active cross-directional pulling, cloacal coupling was maintained without rupture. Upon reaching the opposite bank, the pair crawled onto land, maintaining copulatory union (Figure 2d). Shortly after exiting the water, the female initiated sustained forward crawling, leading to mechanical separation. The male’s everted hemipenis disengaged from the female cloaca (Figure 2e). The female crawled rapidly away into the understory, while the male remained stationary on the ground for an additional 4 min, actively completing the muscular invagination and retraction of his hemipenis into the tail base before retreating (Figure 2f; ESM Video S1). In total, the observed duration of copulatory coupling was approximately 3 h.

### 3.2. Event 2: Diurnal Copulation on Flooded Forest Floor (Rémire-Montjoly)

**Figure 3:**
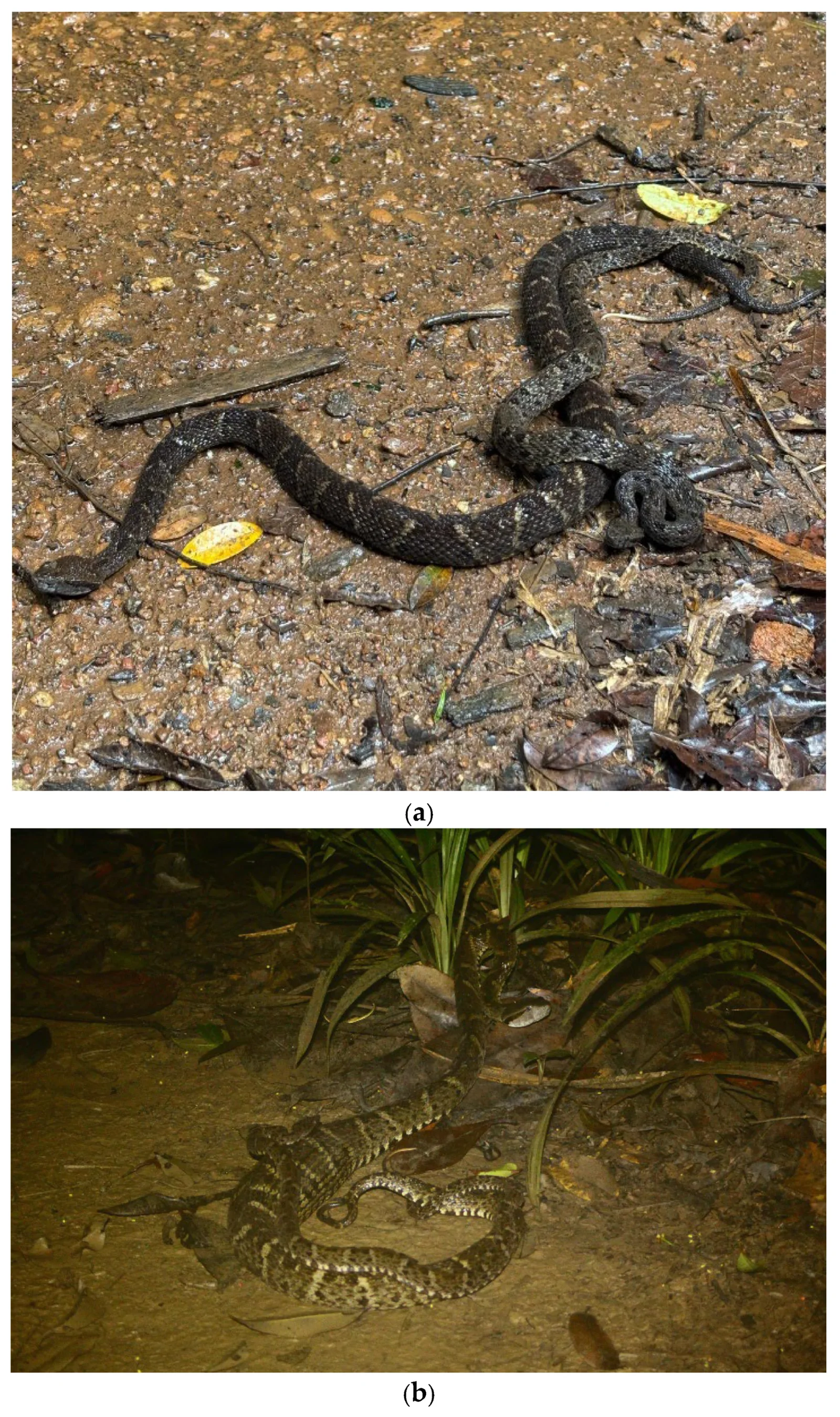
Distinct reproductive interactions of *B. atrox* across wet forest microhabitats. (**a**) Diurnal copulation (Event 2) on a flooded forest trail at Chemin de l’Habitation Vidal, Rémire-Montjoly. (**b**) Nocturnal advanced courtship and attempted copulation (Event 3) at Camp Cariacou, Kourou. The smaller male is seen aligning his body over the larger, recently fed female, executing caudocephalic waves and tail-search behaviors. Because true intromission was not visually confirmed, this event is strictly classified as courtship.

On April 23rd of 2025 at 11:00 h, a second mating event was recorded at Chemin de l’Habitation Vidal, Rémire-Montjoly (Figure 3a; Table 1). An adult pair was found actively in copula on a flooded secondary forest trail under direct daylight. The pair remained stationary in shallow standing water (~5 cm depth) amidst decaying leaves. The female remained motionless in a loose rectilinear posture while the male rested diagonally above her dorsum, maintaining cloacal contact beneath his tail. The pair exhibited no evasive reaction considering the observers, maintaining passive copulatory quiescence during 20 min of observation.

### 3.3. Event 3: Nocturnal Advanced Courtship on Wet Forest Trail (Kourou)

On April 15th of 2026 at 20:33 h, a third reproductive event was recorded at Camp Cariacou, Kourou (Figure 3b; Table 1). Heavy rainfall occurred at the beginning of the night, and the observation was conducted after the rain had subsided, lasting for a period of 15 min. Two *B. atrox* individuals were found actively in advanced courtship and attempted copulation on a wet forest trail. The female had recently fed, as evidenced by a large content in her stomach. The male individual (smaller in body size) positioned himself by aligning his body over the female (larger in body size) (Figure 3b). The male displayed caudocephalic waves and forward movements on the female’s back with intense tongue flicking. The male-initiated tail search attempted copulation to introduce the hemipenis, but true intromission was not verified visually (Figure 3b).

### 3.4. Event 4: Ritualized Male–Male Combat Dynamics (Kourou)

**Figure 4:**
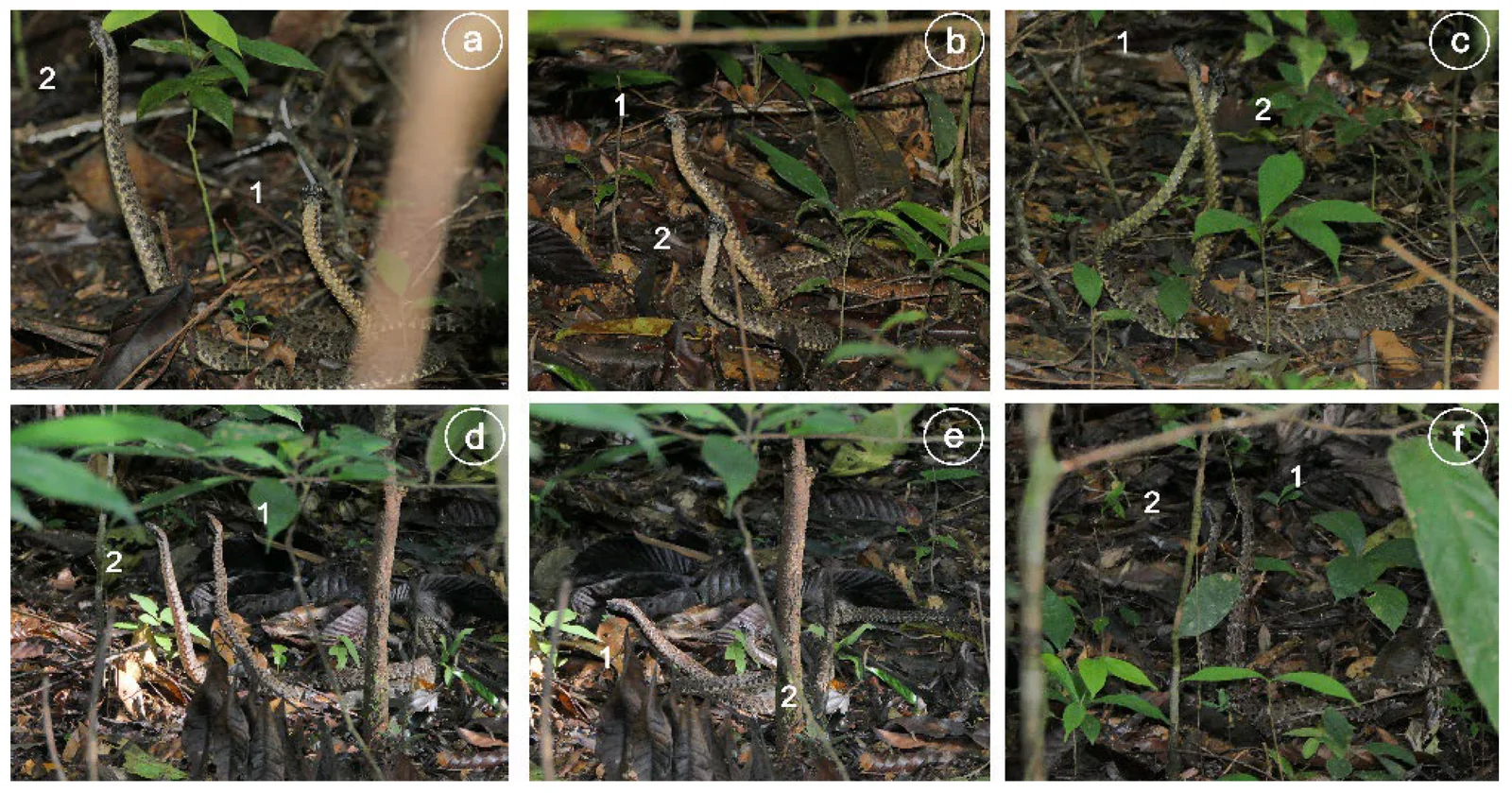
Sequential motor stages of ritualized male–male combat in *Bothrops atrox* (Event 4; Montagne des Singes, Kourou). (**a**,**b**) Stage 1: Vertical postural display and mutual anterior-trunk swaying. (**c**) Stage 2: Anterior entwinement and reciprocal topping attempts, with Male 1 hooking over Male 2. (**d**) Stage 3: Temporary disentanglement and lateral fall to the leaf litter. (**e**,**f**) Stage 4: Re-engagement and posterior-trunk alignment.Numbers 1 and 2 designate the individual male combatants (Male 1 and Male 2, respectively) throughout the interaction sequence. The combatants were continuously distinguished in the field via subtle natural phenotypic variations in their dorsal patterning and slight differences in body size. Given the strictly non-invasive protocol, the sex of the individuals was robustly inferred from these stereotypic male-exclusive crotaline agonistic displays.

On 1 April 2024 between 08:26 and 08:31 h, two adult male *B. atrox* were recorded engaging in ritualized combat on the ground adjacent to a trail at Montagne des Singes, Kourou (Figure 4; Table 1). The interaction lasted approximately 5 min and was documented through a sequential high-resolution photographic series capturing four stereotypic motor stages characteristic of crotaline agonistic bouts (Figure 4):

Stage 1 (Vertical Postural Display and Mutual Swaying): Both males elevated their heads and the anterior third of their bodies into an erect vertical column, facing each other at a distance of 10–20 cm (Figure 4a,b). They performed rhythmic forward-and-backward oscillatory swaying movements of their vertical trunks. Neither snake dealt open-mouth bites or predatory strikes to the other.

Stage 2 (Entwinement and Reciprocal Topping): The males closed distance, entwining their elevated anterior bodies. Male 1 hooked his head and anterior neck over the dorsal neck of Male 2, attempting to force his opponent’s head down against the ground (topping display) (Figure 4c). Male 2 resisted by flexing his body column upwards, displaying a rotational spiral movement between the contestants.

Stage 3 (Disentanglement and Ground Separation): The physical strain of the vertical entwinement caused both combatants to lose equilibrium, tumbling laterally to the leaf litter (Figure 4d). The snakes separated completely for approximately 10 s, orienting parallel to one another under the substrate.

Stage 4 (Re-engagement and Posterior-Trunk Alignment): Both males rapidly elevated their anterior bodies once again, re-establishing physical contact (Figure 4e,f). During this final stage, their posterior trunks were entwined along the ground in tight ventral contact, while the anterior portions remained vertically aligned, with Male 1 consistently holding his head slightly higher than Male 2. Following this bout, Male 2 lowered his body column and crawled into dense root tangles, whereas Male 1 remained in a low horizontal posture on the encounter site displaying slowly dispersing.

## 4. Discussion

### 4.1. Biomechanics of Aquatic Copulation and Locomotor Sexual Conflict

The aquatic copulatory sequence documented in Roura (Video S1) provides rare empirical evidence of bimodal locomotion and locomotor sexual conflict in free ranging pitvipers. In squamates, copulation imposes severe locomotor, energetic, and predation costs [1,10]. In crotalines, copulatory locking is reinforced by specialized hemipenial morphology, which features a bifurcated body adorned with dense proximal basal spines and complex distal calyces that expand within the female cloacal chamber. This mechanical anchoring prevents premature separation by rival males such as physical disturbances during the prolonged coupling phase [14,15].

Regarding the Roura event, our focal video record illustrates how this anatomical lock interacts with sexual size dimorphism (SSD). In *B. atrox*, adult females attain significantly larger body dimensions and mass than conspecific males [7,8] (see Figure 3). When startled or perturbed, the larger female commands total locomotor control of the pair. The female entered the 20 m-wide stream pulling the coupled male as a passive drag (Figure 2c; ESM Video S1). The male’s counter-directional swimming strokes demonstrate an active attempt to anchor himself or break contact, confirming that the movement was entirely female-driven. This represents the first properly documented record of such a bimodal locomotor sequence.

Previous authors have noted that *B. atrox* frequently utilizes seasonally flooded environments, swampy margins, and stream edges for thermoregulation and ambush foraging [8,9]. However, the hypothesis that entering water during mating serves an active foraging function is biologically untenable. First, dietary analyses demonstrate that adult *B. atrox* feeds predominantly on small endothermic mammals, whereas anurans are consumed almost exclusively by juveniles [14,16]. Second, the neurophysiological and mechanical demands of copulatory anchoring, combined with the extreme physical strain of dragging a coupled partner across a water column, fully inhibit predatory strike reflexes and prey ingestion. The maintenance of the copulatory lock under such conditions suggests that the basal spines of the hemipenis might possess sufficient mechanical resilience to withstand the shear forces associated with female-driven locomotion and size dimorphism, although the exact magnitude of these forces remains unquantified. The male completed normal muscular invagination and retraction into the tail base only after returning to land and achieving physical separation (Figure 2e,f; ESM Video S1), in accordance with copulatory timelines observed in crotalines [15,17]. However, the fact that a large female was visibly fed in Kourou did not prevent the female from being attractive and being courted by a male. The female kept her body stretched indicating that she was receptive to male courtship [1,15].

### 4.2. Motor Patterns and Dominance Dynamics in Male–Male Combat

Regarding the male–male combat documented in Kourou (Figure 4), it confirms that the complex agonistic repertoire of *B. atrox* is highly conserved between the western Brazilian Amazon (Acre; [4]) and the eastern Guiana Shield (French Guiana; Table 1), supporting behavioral stasis across distinct lineages of the B. atrox complex [4,18]. All stereotypic behavioral elements described by Fonseca et al. [4]—vertical trunk elevation, horizontal swaying, anterior entwinement, neck hooking, and reciprocal topping—were fully expressed in the Montagne des Singes interaction [19].

Ethologically, male combat in pitvipers is a non-injurious test of physical endurance and leverage [2,6]. While earlier studies often categorized contestants into static “dominant” and “subordinate” roles based solely on which individual maintained a taller vertical column [5,6], our behavioral and photographic analysis indicates that vertical superiority is dynamic and transient. In snakes, genuine behavioral dominance cannot be diagnosed solely by instantaneous height advantages during active fighting; it requires the unambiguous submission of the loser, manifested as flattened body posture, sustained flight, and refusal to re-engage when challenged [2,5,6,20]. In our photographic sequence, although Male 1 maintained a higher posture during the final stage, the combatants had previously collapsed and separated before resuming vertical contests with equal vigor (Figure 4d–f). Thus, we characterize these motor patterns as symmetrical competitive escalation rather than a pre-established social hierarchy.

### 4.3. Implications for Neotropical Pitviper Mating Systems

A geographic synthesis of free-ranging reproductive behavior in *B. atrox* reveals a strong pan-Amazonian pattern. Mating and combat events across the western Brazilian Amazon (Acre), central Amazonia (Manaus), and the eastern Guiana Shield (French Guiana) display a strong probabilistic association with the rainy season (Table 1). This macro-regional synchronization aligns with the vitellogenic cycle and male SSK hypertrophy documented for Amazonian populations [1,7], providing robust empirical support for the prolonged mate-searching polygyny model across the species’ distribution [4]. However, while spermatogenesis and rainfall peak together, studies like [7] have shown that physiological mechanisms such as facultative sperm storage allow for a broader and more plastic mating window, extending to months like May and November. Furthermore, as highlighted in Table 1, these behaviors are recurrently associated with seasonally wet forest floors, flooded trails, as well as riparian margins, displaying the critical role of hydric microhabitats in mediating both mate detection and intersexual interactions. The mating events documented in April corroborate that reproductive activity in free-ranging *B. atrox* is tightly synchronized with the peak of the rainy season [1,7].

Under this system, the spatial distribution of receptive females is widely dispersed and unpredictable, forcing males to travel extensive distances through the forest floor [8,9]. The low frequency of natural combat observations across the species’ range is not an artifact of behavioral absence, but rather an outcome of the brief duration of combat bouts (~5 min in our study; ~5 min in Fonseca et al.) combined with the extreme crypsis and rapid post-contest dispersal of the losers to avoid further agonistic encounters [4,5]. Detailed field documentation, as presented here through focal videography and sequential photography, confirms that male *B. atrox* actively engage in physical contests over reproductive priority in free-ranging conditions.

## 5. Conclusions

In summary, these video insights and field documentations uncover key biomechanical and ethological nuances of reproduction in free-ranging B. atrox. Female-biased sexual size dimorphism confers total locomotor dominance to the female during copulation, enabling pair relocation by aquatic barriers despite active male resistance and without structural failure of the hemipenial anchor under hydrodynamic drag. Additionally, the documented male–male combat demonstrates that ritualized agonistic displays are motorically conserved between western Amazonian and eastern Guiana Shield populations, reflecting behavioral stasis across distinct lineages of the B. atrox populations.

Furthermore, the macro-regional synchronization of mating and combat events during the rainy season across pan-Amazonian populations provides robust empirical support for the prolonged mate-searching polygyny model. Archiving high-resolution focal video records alongside detailed field photographic sequences establishes an essential empirical foundation to refine comparative evolutionary analyses of sexual conflict, functional morphology, and mating systems in Neotropical pitvipers.

## Figures and Tables

**Table 1:** Summary of reproductive behavioral events of the Common Lancehead (*Bothrops atrox*) documented in coastal French Guiana (2024–2026), with corresponding microclimatic conditions and associated documentation format.

Study Site/Region	Date/Period *	Behavior Recorded	Microhabitat/Habitat	Main References
Manaus, Central Brazilian Amazon	April/May/November 1996–2013	Mating (4 observations)	Terrestrial and stream habitats	[7]
Acre, Western Brazilian Amazon	21–23 January 2019	Male–male combat	Wet, seasonally flooded area	[4]
Roura, French Guiana	16 February 2024	Mating	Terrestrial and aquatic; forest stream (*igarapé* ^†^)	Present study
Kourou, French Guiana	1 April 2024	Male–male combat	Wet forest trail	Present study
Rémire-Montjoly, French Guiana	23 April 2025	Mating	Wet/flooded trail	Present study
Kourou, French Guiana	15 April 2026	Advanced courtship- Attempted copulation	Wet forest trail	Present study

* Dates and periods cited for literature records reflect the temporal precision reported in the original publications. ^†^ Small, shallow Amazonian stream or narrow water channel that runs through the rainforest and is traditionally used for canoe navigation.

## Data Availability

The video recordings supporting the findings of this study are available in the Supplementary Material of this article (Video S1). All other relevant behavioral data and photographic series are contained within the manuscript. Additional raw video files and high-resolution photographic series are available from the corresponding author upon reasonable request.

## Supplementary Materials

The following supporting information can be downloaded at: https://www.mdpi.com/article/10.3390/ani16213419/s1, Video S1: Copulatory video sequence.

## Abbreviations

The following abbreviations are used in this manuscript:

a.s.l.	Above sea level
CNPq	Conselho Nacional de Desenvolvimento Científico e Tecnológico
DEAL	Direction de l’Environnement, de l’Aménagement et du Logement
ESM	Electronic Supplementary Material
GPS	Global Positioning System
HACC	Herpetological Animal Care and Use Committee
IMBio	Instituto de Manejo da Biodiversidade
SSD	Sexual size dimorphism
SSK	Sexual segment of the kidney
SVL	Snout-vent length
UNESP	Universidade Estadual Paulista
WGS84	World Geodetic System 1984

## References

[1] Almeida-Santos S.M., Salomão M.G., Schuett G.W., Höggren M., Douglas M.E., Greene H.W. (2002). Reproduction in Neotropical pitvipers, with emphasis on species of the genus *Bothrops*. Biology of the Vipers.

[2] Carpenter C.C. (1977). Communication and displays of snakes. Am. Zool..

[3] Greene H.W. (1997). Snakes: The Evolution of Mystery in Nature.

[4] Fonseca W.L., Correa R.R., Oliveira A.S., Braz H.B., Almeida-Santos S.M., Bernarde P.S. (2022). First report of male–male combat in free-ranging Amazonian Common Lanceheads (*Bothrops atrox*). Can. J. Zool..

[5] Carpenter C.C., Seigel R.A., Hunt L.E., Knight J.L., Malaret L., Zuschlag N.L. (1984). Dominance in snakes. Vertebrate Ecology and Systematics: A Tribute to Henry S. Fitch.

[6] Schuett G.W. (1997). Body size and agonistic experience affect dominance and mating success in male copperheads. Anim. Behav..

[7] Silva K.B., Silva K.M.P., Sueiro L.R., Oliveira M.E.E.S., Almeida-Santos S.M. (2019). Reproductive biology of *Bothrops atrox* (Serpentes, Viperidae, Crotalinae) from the Brazilian Amazon. Herpetologica.

[8] Oliveira M.E., Martins M. (2001). When and where to find a pitviper: Activity patterns and habitat use of the lancehead, *Bothrops atrox*, in central Amazonia, Brazil. Herpetol. Nat. Hist..

[9] Turci L.C.B., Albuquerque S., Bernarde P.S., Miranda D.B. (2009). Activity, habitat use, and behavior of *Bothriopsis bilineatus* and *Bothrops atrox* (Serpentes: Viperidae) in Moa River Forest, Acre—Brazil. Biota Neotrop..

[10] Duvall D., Arnold S.J., Schuett G.W., Campbell J.A., Brodie E.D. (1992). Pitviper mating systems: Ecological potential, sexual selection, and microevolution. The Biology of Pitvipers.

[11] Nogueira C.C., Argôlo A.J.S., Arzamendia V., Azevedo J.A., Barbo F.E., Bérnils R.S., Centeno F.C., Columbiano B.F., Costa F.C., Fraga R. (2019). Atlas of Brazilian snakes: Verified point-locality maps to mitigate the Wallacean shortfall in a megadiverse snake fauna. S. Am. J. Herpetol..

[12] Silva K.M.P., Breno M.C., Sueiro L.R., Sant’Anna S.S., Almeida-Santos S.M. (2013). Reprodução de *Bothrops* spp. (Serpentes, Viperidae) em criadouro conservacionista. Vet. Zootec..

[13] Altmann J. (1974). Observational study of behavior: Sampling methods. Behaviour.

[14] Bisneto P.F., Kaefer I.L. (2019). Reproductive and feeding biology of the common lancehead *Bothrops atrox* (Serpentes, Viperidae) from central and southwestern Brazilian Amazonia. Acta Amaz..

[15] Garani Y., Costa H.C., Rodrigues-Neto A.C., Duarte S.J., Almeida-Santos S.M. (2024). First record of courtship and mating in Neuwied’s Lancehead, *Bothrops neuwiedi* Wagler, 1824 in nature. Herpetol. Notes.

[16] Martins M., Marques O.A.V., Sazima I., Schuett G.W., Höggren M., Douglas M.E., Greene H.W. (2002). Ecological and phylogenetic correlates of feeding habits in Neotropical pitvipers of the genus *Bothrops*. Biology of the Vipers.

[17] Almeida-Santos S.M., Santos T., Lobo L.M. (2021). Mating in free ranging Neotropical rattlesnakes, *Crotalus durissus*: Is it risky for males?. Herpetol. Notes.

[18] Wüster W., Thorpe R.S., Puorto G., Furtado M.F.D., Hoge S.A., Salomão M.G., Theakston R.D.G., Warrell D.A. (1996). Systematics of the *Bothrops atrox* complex (Reptilia: Serpentes: Viperidae) in Brazil: A multivariate analysis. Herpetologica.

[19] Carpenter C.C., Gillingham J.C., Murphy J.B. (1976). The combat ritual of the rock rattlesnake (*Crotalus lepidus*). Copeia.

[20] Batista S.F., Muniz-da-Silva D.F., Almeida-Santos S.M. (2021). Dominant and submissive behaviour in the rattlesnake *Crotalus durissus* under semi-natural conditions. Herpetol. Bull..

